# Novel catalytic properties of quadruple perovskites

**DOI:** 10.1080/14686996.2017.1350557

**Published:** 2017-07-27

**Authors:** Ikuya Yamada

**Affiliations:** ^a^ Department of Materials Science, Graduate School of Engineering, Osaka Prefecture University, Sakai, Japan

**Keywords:** Quadruple perovskite, catalysis, high-pressure synthesis, 50 Energy Materials, 206 Energy conversion / transport / storage / recovery, 207 Fuel cells / batteries / super capacitators, 504 X-ray / Neutron diffraction and scattering, 107 Glass and ceramic materials, 205 Catalyst / photocatalyst / photosynthesis

## Abstract

Quadruple perovskite oxides *AA*′_3_ *B*_4_O_12_ demonstrate a rich variety of structural and electronic properties. A large number of constituent elements for *A*/*A*′/*B*-site cations can be introduced using the ultra-high-pressure synthesis method. Development of novel functional materials consisting of earth-abundant elements plays a crucial role in current materials science. In this paper, functional properties, especially oxygen reaction catalysis, for quadruple perovskite oxides CaCu_3_Fe_4_O_12_ and *A*Mn_7_O_12_ (*A* = Ca, La) composed of earth-abundant elements are reviewed.

## Graphical abstract

**Figure:**
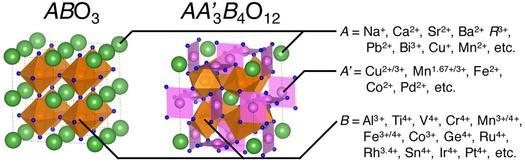

## 1. Introduction

**Figure 1.**
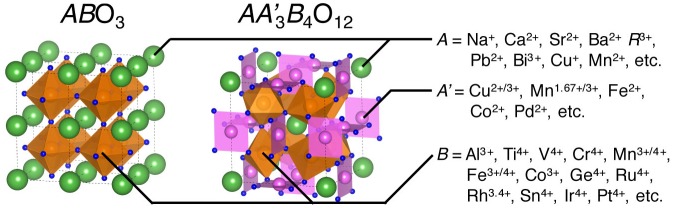
Crystal structure of simple (*AB*O_3_-type) and quadruple (*AA*′_3_ *B*_4_O_12_-type) perovskites.

Perovskite oxides *AB*O_3_ (*A* = alkaline, alkaline-earth and rare-earth metal ions; *B* = d-block transition metals) have been extensively investigated as promising functional materials in the past decades [1–3]. A large number of perovskite and related materials were synthesized and their properties were exhaustively illustrated. Partial chemical substitutions of *A*- and/or *B*-sites for *AB*O_3_-type perovskite and related structures drive structural and electronic transformations, leading to functional properties such as large magnetoresistance and high-temperature superconductivity [4,5]. A couple of different metal ions occupying the identical crystallographic sites make spatial ordering of atoms, crystallizing in ordered perovskite structures with multiplied chemical formulae such as *A*_2_ *BB*′O_6_, *AA*′*B*_2_O_6_ and *AA*′_3_ *B*_4_O_12_. Sr_2_FeMoO_6_ is a double perovskite consisting of two *B*-site ions, Fe^3+^ and Mo^5+^, in which a significant difference in valence states results in rock-salt type ordering of these two ions [6]. YBaMn_2_O_6_ is another type of double perovskites with layered ordering of *A*-site ions Y^3+^ and Ba^2+^ [7]. The quadruple perovskite oxide family *AA*′_3_ *B*_4_O_12_ is derived from 1:3-type *A*-site cation ordering; one quarter (*A*) is occupied by conventional *A*-site ions in icosahedral coordination, the remaining three quarters by transition metal ions in pseudosquare-planar coordination (Figure 1). The quadruple perovskite oxide family was discovered by Bochu et al. in the 1970s [8,9], and has now been intensively investigated because of a lot of syntheses of new compounds using ultra high pressures above 10 GPa. Combinations of *A*-, *A*′- and *B*-site ions provide unexpected properties, some of which are functional. In this paper, recent advances on the catalytic properties of the quadruple perovskite oxides, especially those consisting of earth-abundant elements, are reviewed.

## 2. Quadruple perovskite oxides

### 2.1. Syntheses, compositions and crystal structures

Most quadruple perovskite oxides *AA*′_3_ *B*_4_O_12_ crystallize in the cubic symmetry of *Im* 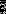 space group (No. 204), containing smaller transition metal ions at pseudosquare-coordinated *A*′-sites. High-pressure syntheses under several GPa are usually adopted to stabilize smaller *A*′-site ions at sites with an originally high coordination number of 12. Ultra-high-pressure syntheses above 10 GPa efficiently expand the variety of *A*′-site cations. Together with Jahn-Teller active ions with 3d^4^ and 3d^9^ electron configurations Mn^3+^ and Cu^2+^, respectively [8,9], non-Jahn-Teller ions such as Mn^1.67+^, Fe^2+^, Co^2+^, Cu^3+^ and Pd^2+^ can be incorporated [10–14]. Tri- and tetra-valent *B*-site cations in this series are reported: Al^3+^, Ti^4+^, V^4+^, Cr^3+/4+^, Mn^3+/4+^, Fe^3+/4+^, Co^3+^, Ge^4+^, Ru^4+^, Rh^3.4+^, Sn^4+^, Ir^4+^ and Pt^4+^ [9,15–23]. The icosahedral *A*-sites are occupied by conventional alkaline (Na^+^, K^+^), alkaline-earth (Ca^2+^, Sr^2+^, Ba^2+^) and rare-earth (*R* ^3+^: *R* = La–Lu, Ce^4+^) metal ions, and also by Cd^2+^, Pb^2+^, Th^4+^, Bi^3+^, Ag^+^ [24–26]. Unusual *A*-site ions in this series are Mn^2+^ and Cu^+^ ions for *A*Cu_3_V_4_O_12_ [27,28], and the most remarkable example is ζ-Mn_2_O_3_ (MnMn_3_Mn_4_O_12_) [29]. Recently, quadruple perovskite PbCoO_3_ (Pb^2+^Pb^4+^_3_Co^2+^_2_Co^3+^_2_O_12_), in which both *A*- and *B*-site aliovalent ions are simultaneously ordered, has been reported [30]. Several examples with *B*-site ordering of a different kind of cations in *Pn* 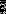 symmetry (space group No. 201) are also known: CaCu_3_Ga_2_Sb_2_O_12_, CaCu_3_Cr_2_Sb_2_O_12_ and CaCu_3_Fe_2_ *B*′_2_O_12_ (*B*′ = Re, Sb, Nb, Os) [31–37].

### 2.2. Electronic properties

Electronic properties of quadruple perovskite oxides have been widely investigated. CaCu_3_Ti_4_O_12_ is the best-known quadruple perovskite and is investigated as a dielectric material owing to its large permittivity [38,39]. (Ca/La)Cu_3_Mn_4_O_12_ exhibits a room-temperature magnetoresistance in low magnetic fields [40,41]. CaCu_3_Ru_4_O_12_ and CaCu_3_Ir_4_O_12_ show enhanced electron mass, like a heavy fermion [42,43]. NaMn_7_O_12_ displays sequential charge/spin/orbital orderings [44]. These properties are summarized in the review [45].

The above-mentioned properties are mainly attributed to electronic interactions between *A*′- and *B*-site metals. A striking feature of *A*′–*B* site electronic interactions is intermetallic charge transfer reported for the *A*Cu_3_Fe_4_O_12_ system. A temperature-induced *A*′-to-*B* electron charge transfer from Cu to Fe (3Cu^2+^ + 4Fe^3.75+^ → 3Cu^3+^ +4Fe^3+^) occurs at ~400 K for LaCu_3_Fe_4_O_12_ [13], although a charge disproportionation (2Fe^4+^ → Fe^3+^ +Fe^5+^) below 210 K was reported for Ca-analogue CaCu_3_Fe_4_O_12_ previously [17]. Later, an inverse *B*-to-*A*′ electron charge transfer from Fe to Cu simultaneously with the charge disproportionation was demonstrated for CaCu_3_Fe_4_O_12_ [46], ensuring the bidirectionality of *A*′-*B* intersite charge transfer. The charge-disproportionation/transfer transitions are abruptly switched by bond strains on *R*–O (*R*: rare-earth metals) and Fe–O bonds for *R*Cu_3_Fe_4_O_12_ [47,48], evidencing multiple competing electronic states in *A*Cu_3_Fe_4_O_12_ series [49].

**Figure 2.**
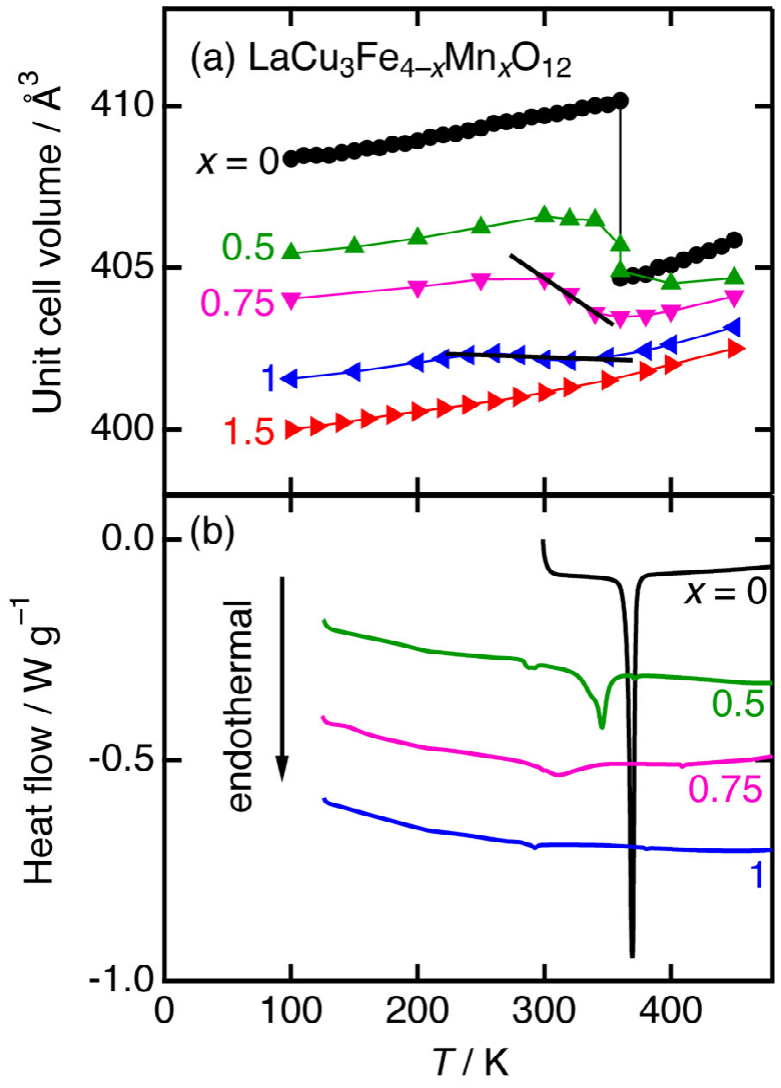
Temperature dependence of the unit cell volume (a) and differential scanning calorimetry curves (b) of LaCu_3_Fe_4–*x*_Mn_*x*_O_12_ (*x* = 0, 0.5, 0.75, 1, and 1.5). Reproduced from [53] with permission from AIP Publishing LLC.

**Figure 3.**
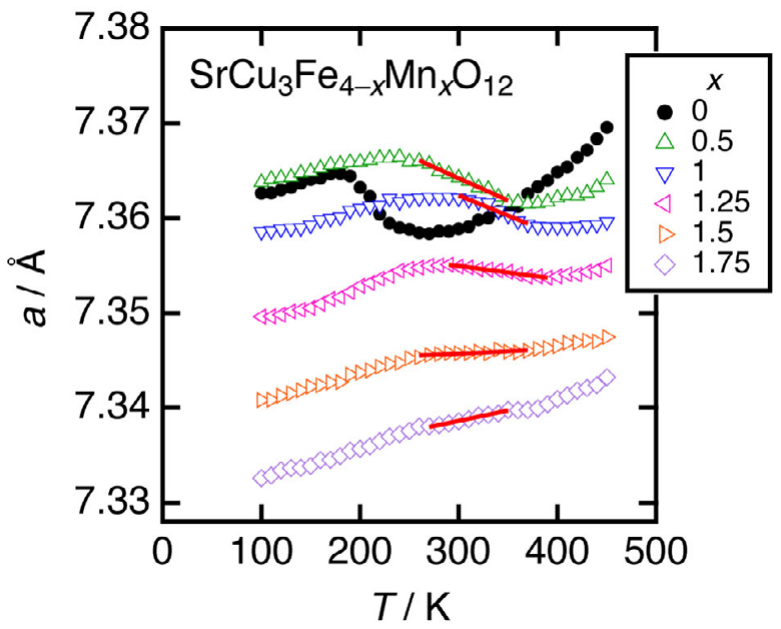
Temperature dependence of the cubic *a*-axis length for SrCu_3_Fe_4–*x*_Mn_*x*_O_12_ (*x* = 0, 0.5, 1, 1.25, 1.5, and 1.75). Reprinted from [54] with permission from AIP Publishing LLC.

SrCu_3_Fe_4_O_12_ displays a second-order continuous Cu-to-Fe electron charge transfer associated with a negative thermal expansion in the temperature range between ~200 and ~270 K [50–52]. More recently, adjustment of thermal expansion properties, that is, relaxations from first- to second-order charge transfer resulting in negative or near-zero thermal expansions in moderate temperature ranges were found for Mn-doped LaCu_3_Fe_4_O_12_ (Figure 2) [53]. Further, precise control of the thermal expansion coefficient and operation temperature range of negative/zero thermal expansion is achieved for Mn-doped SrCu_3_Fe_4_O_12_ (Figure 3) [54].

### 2.3. Catalytic properties

**Figure 4.**
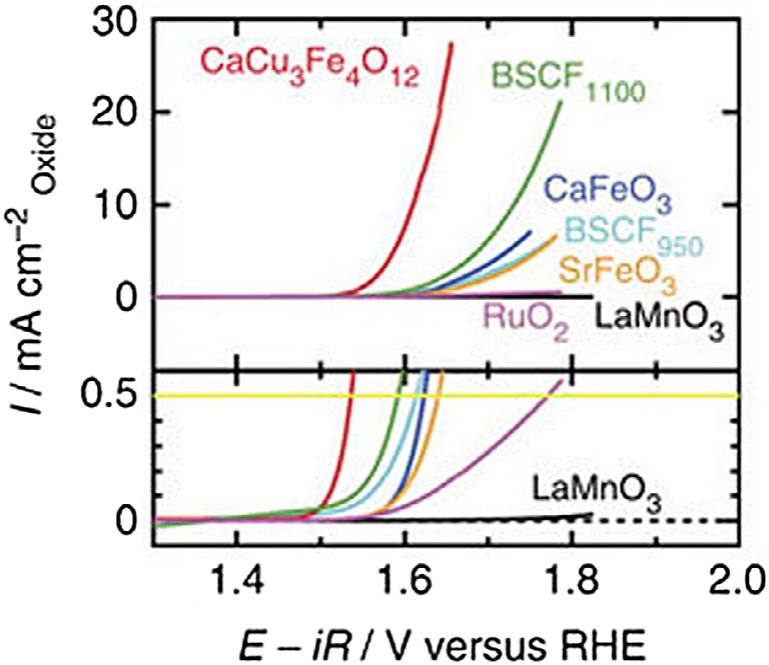
Linear sweep voltammograms in OER conditions for perovskite oxide and RuO_2_ catalysts. Reproduced from [55].

Recently, catalytic properties of quadruple perovskite oxides synthesized under high pressure were reported. Fe^4+^-containing perovskites, CaFeO_3_, SrFeO_3_ and CaCu_3_Fe_4_O_12_ display catalytic activities for the oxygen evolution reaction (OER; 4OH^–^ → O_2_+2H_2_O+4e^–^ in alkaline conditions). These are comparable to or exceed the activities of state-of-the-art OER catalysts such as Ba_0.5_Sr_0.5_Co_0.8_Fe_0.2_O_3–δ_ (BSCF) [55] and RuO_2_ (Figure 4). OER plays a crucial role in energy conversions such as water splitting and recharge of metal–air batteries, as well as the importance of the oxygen reduction reaction (ORR; O_2_+2H_2_O+4e^–^ → 4OH^–^ in alkaline conditions) for fuel cell operations [56–58]. CaCu_3_Fe_4_O_12_ is a promising OER catalyst because of the earth-abundant constituent elements (Ca, Cu and Fe).

The origin of highly active OER catalysis of Fe^4+^-perovskites could be explained by two factors: metal–oxygen covalency and electronic states. Suntivich et al. [59] exhibited a volcano-shaped plot of OER catalytic activity for *AB*O_3_-type perovskites, in which the activity is highest in the vicinity of the e_g_^~1.2^ orbital occupancy of *B*-site ions. Furthermore, Grimaud et al. [60] reported that OER activities of Co-containing double perovskites *Ln*BaCo_2_O_6–δ_ (*Ln* = lanthanide metals) are related to the energy levels of oxygen 2p band center; the OER activity is enhanced when oxygen 2p band center is moderately close to the Fermi level. These tendencies are explained by the OER reaction model in which charge transfer from adsorbed reactants and intermediates to transition metal ions (active sites) of a catalyst is facilitated to decrease the energy barrier of a rate-determining step when the metal–oxygen covalency becomes strong [61]. The unusual high valence ions (Fe^4+^) may form strong covalency with oxygen ions [62], thus the high OER activity is expected for Fe^4+^-perovskites.

**Figure 5.**
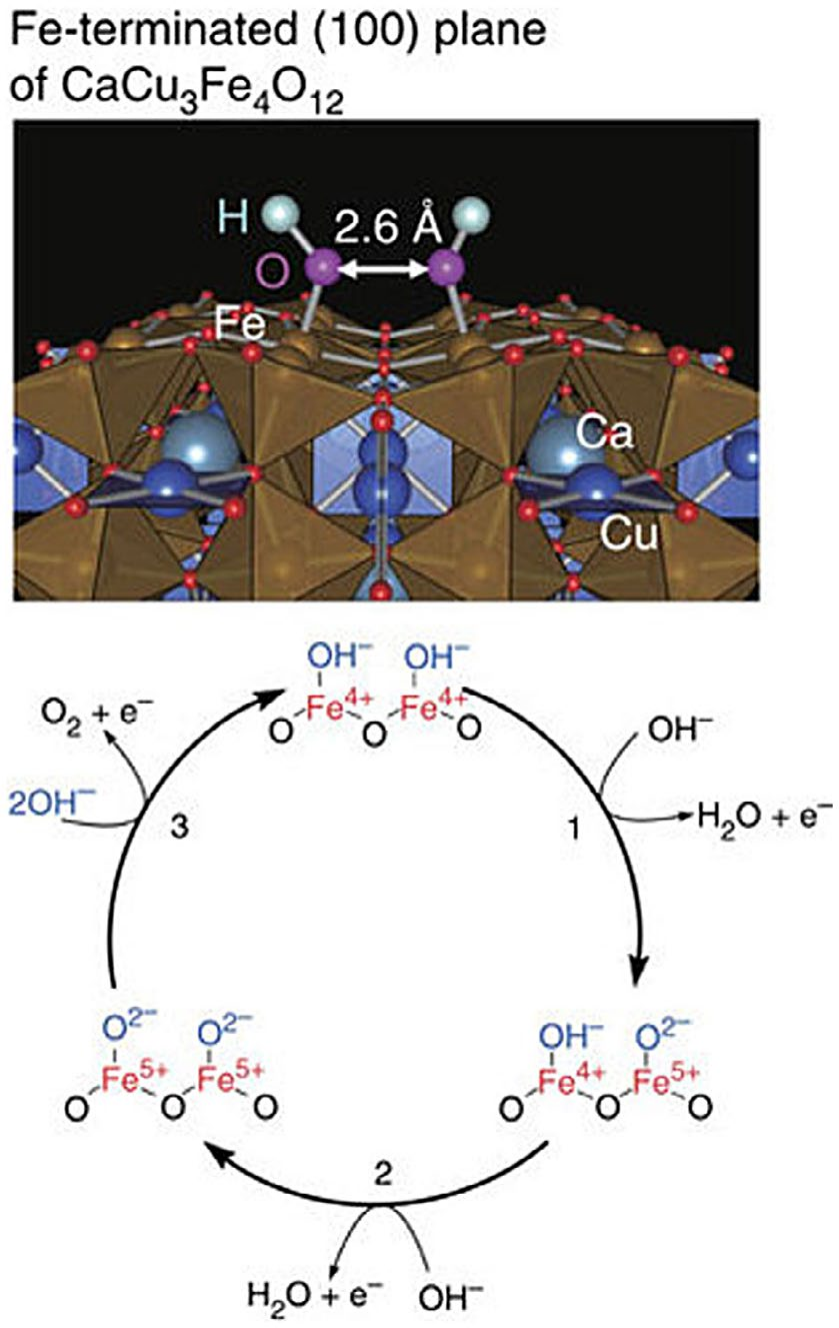
(Top) OH^−^ adsorbates on FeO_2_-terminated (100) planes of CaCu_3_Fe_4_O_12_. The interatomic distance between the nearest neighboring OH adsorbates is ∼2.6 A. (Bottom) Proposed OER reaction mechanism for CaCu_3_Fe_4_O_12_. Reproduced from [55].

More interestingly, CaCu_3_Fe_4_O_12_ is more active for OER than simple perovskite counterparts. Since high OER activity is not known for cupric oxides, the Cu ions incorporated into *A*′-sites are not expected to contribute to the OER activity. In addition, synergistic effects between Cu and Fe do not seem to be achieved because another Cu-Fe complex oxide (spinel-type CuFe_2_O_4_) has a lower activity. Crystal structure analysis suggests that heavily tilted Fe–O–Fe bonds make the neighboring adsorbates close enough to interact, possibly enabling two active-site reaction mechanisms (Figure 5). This mechanism is different from conventional single-active-site reactions for simple perovskite catalysts and is expected to avoid rate-determining steps of conventional mechanisms [59].

**Figure 6.**
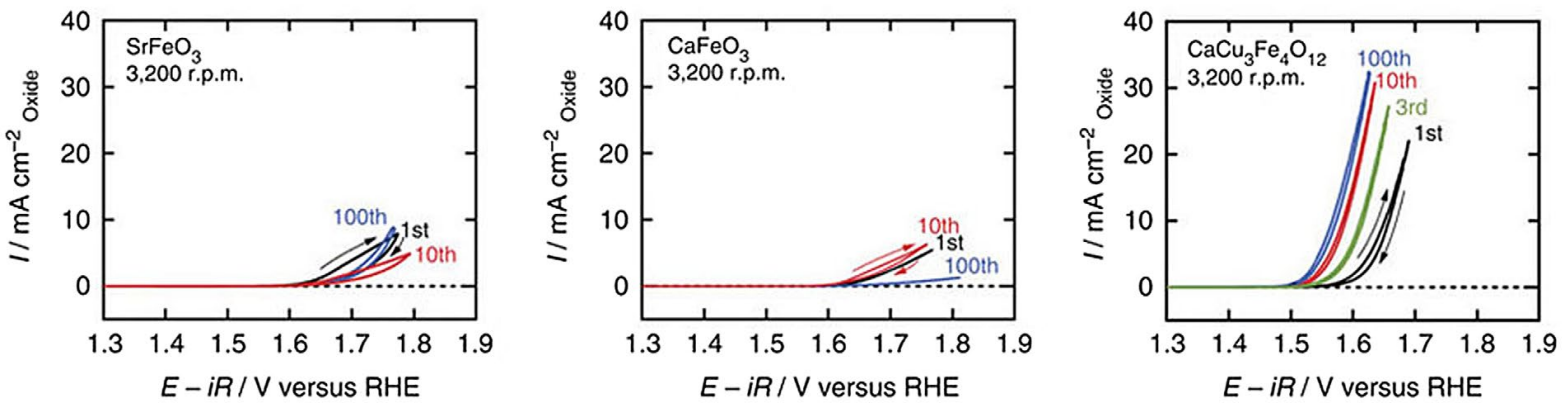
Cyclic voltammograms of SrFeO_3_, CaFeO_3_, and CaCu_3_Fe_4_O_12_ for 100 sequential OER measurements. Reproduced from [55].

**Figure 7.**
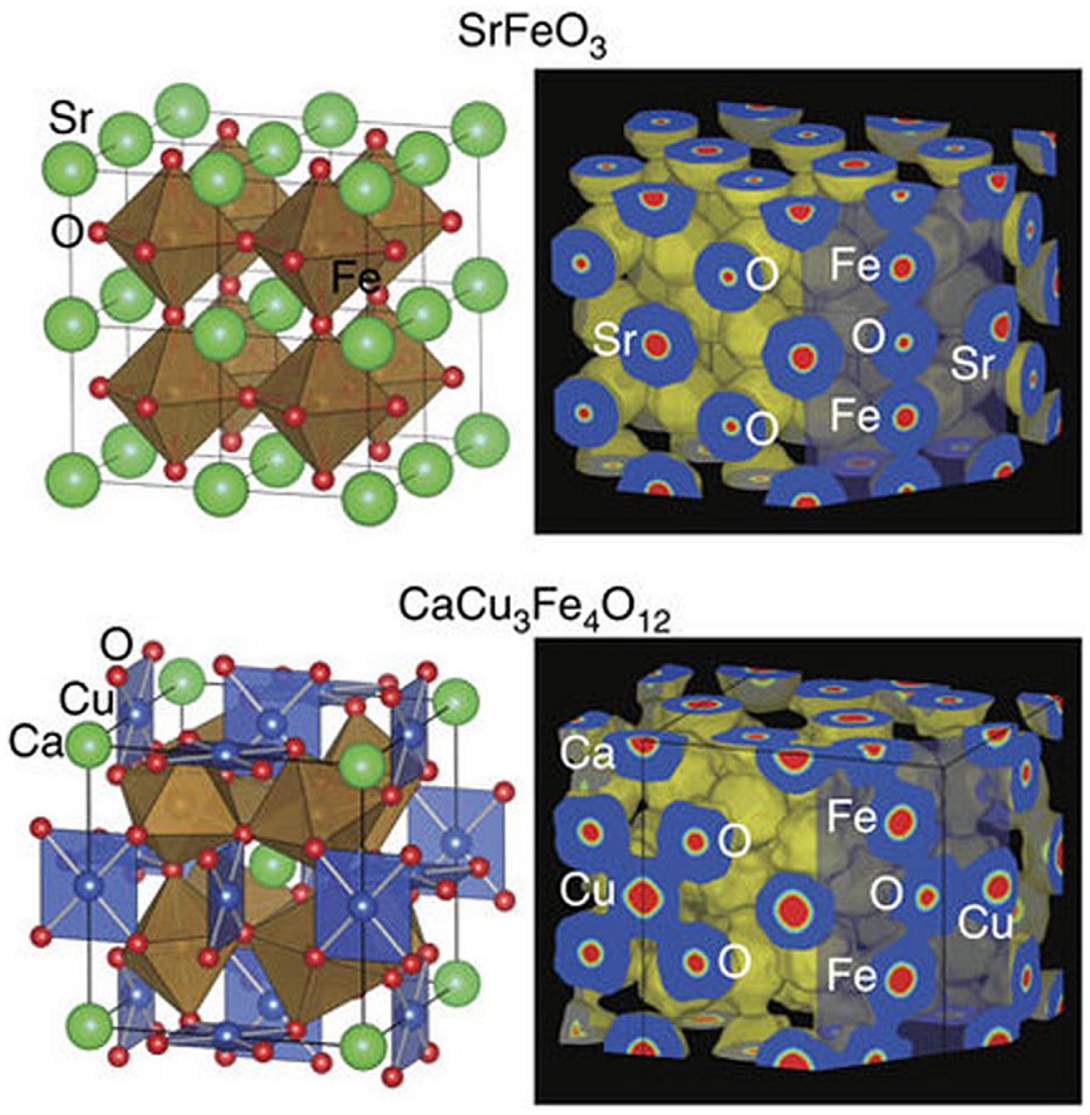
(Left) Crystal structures and (right) electron density maps of SrFeO_3_ (equal-density level: 0.4 Å^−3^) and CaCu_3_Fe_4_O_12_ (equal-density level: 0.5 Å^−3^). Reproduced from [55].

Another remarkable feature of CaCu_3_Fe_4_O_12_ catalyst is durability in OER conditions. The simple perovskites CaFeO_3_ and SrFeO_3_ were readily degraded during 100 sequential OER measurements because of progressive surface amorphization (Figure 6). In contrast, CaCu_3_Fe_4_O_12_ retained the initial OER activity after 100 OER measurements. The enhancement of stability for CaCu_3_Fe_4_O_12_ is attributed to the widespread covalent bonding network consisting of Cu–O and Fe–O bonds. Figure 7 illustrates the electron density distributions of SrFeO_3_ and CaCu_3_Fe_4_O_12_ obtained from Rietveld refinement and maximum entropy method analysis of synchrotron X-ray diffraction data. It is expected that highly ionic Sr ions with electronic isolation from coordinating oxygen ions are easily dissolved in the electrolyte for SrFeO_3_. On the other hand, CaCu_3_Fe_4_O_12_ has a widespread covalent bonding network in which pseudosquare-planar CuO_4_ and octahedral FeO_6_ units are electronically connected. The degradation of perovskite oxide OER catalysts is an essential issue [60,63,64] but efficient strategies have not been established yet. This finding provides a new design principle for highly active and robust catalysts for OER.

**Figure 8.**
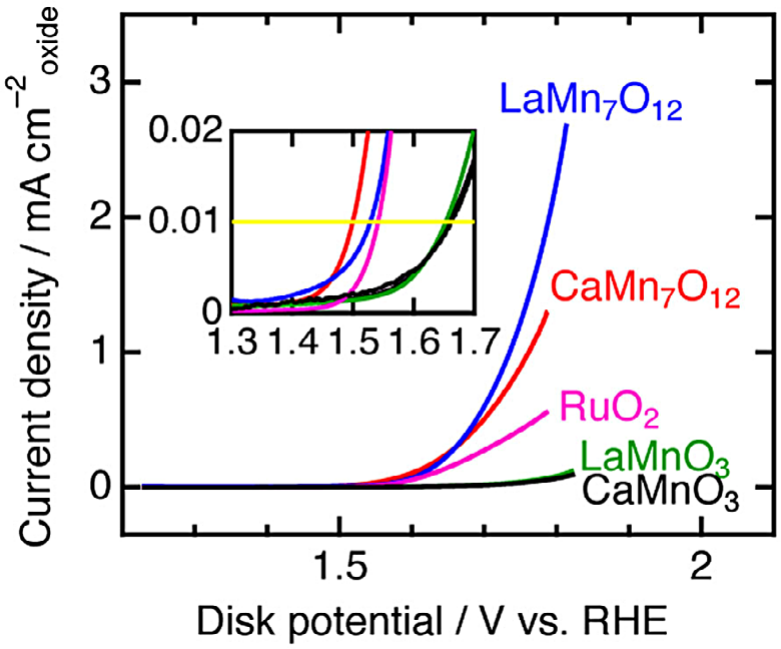
Linear sweep voltammograms in OER conditions for *A*MnO_3_, *A*Mn_7_O_12_ (*A* = Ca, La), and RuO_2_. The inset illustrates the enlarged data in the vicinity of the current density onset. Reproduced from [65] with permission from John Wiley & Sons.

**Figure 9.**
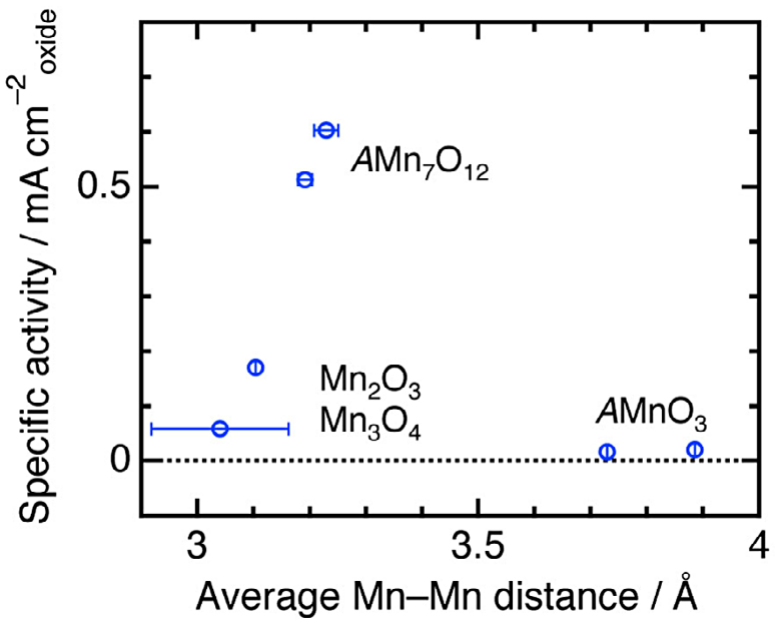
The specific activities versus average Mn–Mn intermetallic distances calculated for edge-shared MnO_6_ octahedra (Mn_2_O_3_, Mn_3_O_4_), corner-shared MnO_4_ pseudosquare plane–MnO_6_ octahedron (CaMn_7_O_12_, LaMn_7_O_12_), and corner-shared MnO_6_ octahedra (CaMnO_3_, LaMnO_3_). Reproduced from [65] with permission from John Wiley & Sons.

**Figure 10.**
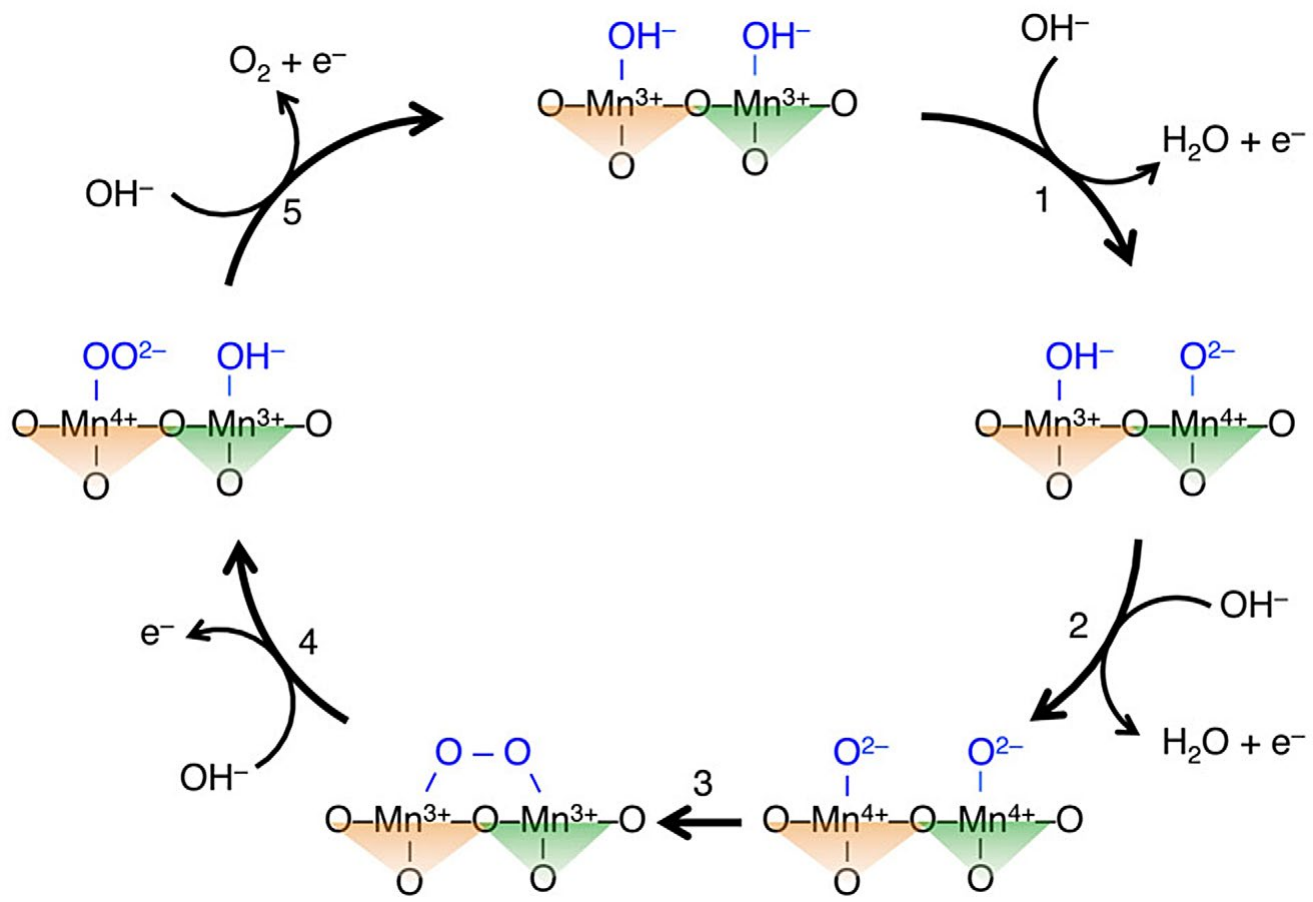
Proposed OER mechanism for LaMn_7_O_12_ via direct O–O bond formation between the unsaturated MnO_4_ plane (green, right triangle) and the unsaturated MnO_6_ octahedron (orange, left triangle). Reproduced from [65] with permission from John Wiley & Sons.

**Figure 11.**
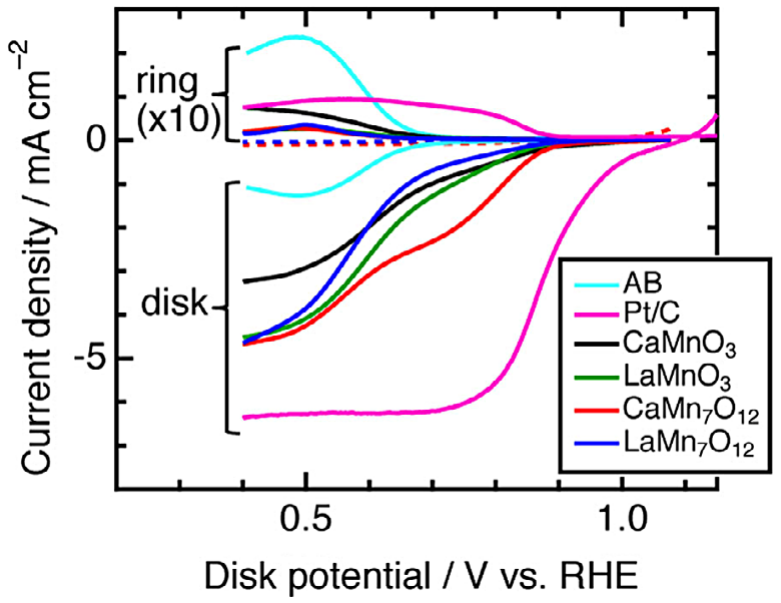
Catalytic activity of manganese perovskites obtained by using a rotating ring/disk electrode equipment. Disk/ring current densities are plotted as a function of applied disk potential in ORR conditions for *A*MnO_3_, *A*Mn_7_O_12_ (*A* = Ca, La), and reference catalysts (acetlylene black (AB), Platinum-carbon composite (Pt/C)). Reproduced from [65] with permission from John Wiley & Sons.

To elucidate *A*′-site cation contributions to a reaction mechanism, comparison of simple and quadruple perovskite catalysts with identical elements/ions was conducted [65]. Since Mn-based perovskites *A*MnO_3_ and *A*Mn_7_O_12_ (*A* = Ca, La) consist of identical elements (Ca, Mn, O) for *A* = Ca or identical ions (La^3+^, Mn^3+^, O^2–^), compositional differences are suppressed, compared with the above-described couple (CaFeO_3_ and CaCu_3_Fe_4_O_12_). Figure 8 shows linear sweep voltammograms of Mn-perovskites in OER conditions. There are clear differences in catalytic activity between *A*MnO_3_ and *A*Mn_7_O_12_ series, namely, overpotentials are lower by 0.1 V and specific activities at 1.7 V versus reversible hydrogen electrode (RHE) are about thirtyfold for *A*Mn_7_O_12_. This observation implies higher catalytic activities of quadruple perovskite oxides are derived not from electronic factors but structural effects altering reaction mechanisms. Indeed, first-principle calculations do not explain the origin of increased OER activity for quadruple manganese perovskites. In contrast, structure analysis based on Rietveld refinement of synchrotron X-ray diffraction data suggests a structure-activity relationship for Mn-oxide catalysts. Figure 9 shows the specific activity versus average Mn–Mn distances for various manganese oxide catalysts including corner/edge-shared Mn–O polyhedral units, in analogy with the Mn–Mn distance dependence of photocatalytic turnover frequency for various manganese oxides [66]. The specific activity is enhanced as the Mn–Mn distance increases from ~3 to ~3.2 Å, although poorly active for longer Mn–Mn distances (~3.8 Å). This feature is explained by possible O–O bond formation for a two-site reaction mechanism. Figure 10 illustrates the proposed OER mechanism for quadruple perovskite catalysts, in which the two neighboring active sites (*A*′- and *B*-site Mn atoms) are close enough to connect their adsorbates. Rate-determining steps including O–O bond formation are avoided in this mechanism [59]. *A*Mn_7_O_12_ catalysts also catalytically active for ORR, as well as most manganese oxides (Figure 11). Thus, *A*Mn_7_O_12_ perovskites are bifunctional catalysts for oxygen reactions (OER and ORR).

## 3. Conclusions

Recent advances on novel functional properties for quadruple perovskite oxides are reviewed. Highly active oxygen reaction catalysis for quadruple perovskite oxides consisting of earth-abundant elements demonstrates that utilization of ultra-high-pressure synthesis facilitates development of novel functional materials. A great number of still-unexplored compounds synthesized under high pressure also remain promising candidates for functional materials. Thus, further investigation of high-pressure-synthesized materials can give new impetus to materials science.

## Disclosure statement

No potential conflict of interest was reported by the author.

## Funding

This work was supported by Grants-in-Aid for Scientific Research [16H04220; 16H02393] from the Japan Society for the Promotion of Science, Toray Science Foundation, and Kyoto Technoscience Center.
